# Signaling network between the dysregulated expression of microRNAs and mRNAs in propofol-induced developmental neurotoxicity in mice

**DOI:** 10.1038/s41598-018-32474-3

**Published:** 2018-09-21

**Authors:** Congshan Jiang, Sarah Logan, Yasheng Yan, Yasuyoshi Inagaki, Thiago Arzua, Peizhong Ma, Shemin Lu, Zeljko J. Bosnjak, Xiaowen Bai

**Affiliations:** 1 Department of Anesthesiology, Medical College of Wisconsin, Milwaukee, WI USA; 2 Department of Biochemistry and Molecular Biology, School of Basic Medical Science, Xi’an Jiaotong University Health Science Center, Xi’an, Shaanxi China; 3 Department of Cell Biology, Neurobiology & Anatomy, Medical College of Wisconsin, Milwaukee, WI USA; 4 Department of Physiology, Medical College of Wisconsin, Milwaukee, WI USA; 5 Department of Medicine, Medical College of Wisconsin, Milwaukee, WI USA

## Abstract

Mounting evidence has demonstrated that general anesthetics could induce acute neuroapoptosis in developing animals followed by long-term cognitive dysfunction, with the mechanisms remaining largely unknown. The aim of this study was to investigate the effect of the intravenous anesthetic propofol on the profiles of microRNAs (miRNAs) and messenger RNAs (mRNAs), and their interactive signaling networks in the developing mouse hippocampus. Postnatal day 7 (P7) mice were exposed to propofol for 3 hours. Hippocampi were harvested from both P7 (3 hours after exposure) and P60 mice for the analysis of the expression of 726 miRNAs and 24,881 mRNAs, and apoptosis. Long-term memory ability of P60 mice was analyzed using the Morris Water Maze. Propofol induced acute apoptosis in the hippocampus, and impaired memory function of mice. There were 100 altered mRNAs and 18 dysregulated miRNAs in the propofol-treated hippocampi compared with the intralipid-treated control tissues on P7. Bioinformatics analysis of these abnormally expressed genes on P7 indicated that 34 dysregulated miRNA-mRNA target pairs were related to pathological neurological and developmental disorder processes such as cell viability, cell morphology and migration, neural stem cell proliferation and neurogenesis, oligodendrocyte myelination, reactive oxygen species, and calcium signaling. Neonatal propofol exposure also resulted in the abnormal expression of 49 mRNAs and 4 miRNAs in P60 mouse hippocampi. Specifically, bioinformatics analysis indicates that among these dysregulated mRNAs and miRNAs, there were 2 dysregulated miRNA-mRNA targets pairs (Fam46a/miR-363-3p and Rgs3/miR-363-3p) that might be related to the effect of propofol on long-term cognitive function. Collectively, our novel investigation indicates that acute and long-term dysregulated miRNA-mRNA signaling networks potentially participate in propofol-induced developmental neurotoxicity.

## Introduction

Mounting evidence from animal studies has shown that various anesthetics exert adverse consequences on developing neuronal cells, resulting in persistent cognitive dysfunction^1–4^. Anesthetic-induced developmental neurotoxicity includes neuronal apoptosis^4^, neurodegeneration^3^, alteration of neurogenesis^5^ and synaptogenesis^6^, and brain circuit impairment^7,8^. Propofol is one of the most commonly used intravenous anesthetic agents in pediatric populations. It was found that even subanesthetic doses of propofol can lead to neuronal cell death in the infant mouse brain^9^, in addition to brain injury in both fetal and neonatal non-human primates^10^. Propofol also altered calcium homeostasis, caused mitochondrial dysfunction, neuroinflammation, and neurotrophin expression deregulation^11,12^, though the detailed mechanism remains largely unknown.

microRNAs (miRNAs) are small non-coding RNAs with 18–23 nucleotides and have been implicated in nearly every physiological process. After their transcription and initial processing in the nucleus, miRNAs are exported to the cytoplasm for final processing. miRNAs negatively regulate their target mRNA by complementary binding of their seed sequence to the target mRNAs^13^. As a result, the target mRNA is degraded or the process of translation is repressed^14^. Abnormal expression of miRNAs has been found to play crucial roles in various pathological processes such as Alzheimer’s, Huntington’s and Parkinson’s disease^15^. Despite their broad biological involvement and challenges to elucidate specific function and mechanisms, miRNAs have become a valuable tool for basic scientists and clinicians for gene interactions, screening processes, and disease prediction^16,17^.

Several studies implicate the importance of microRNAs in propofol-induced developmental neurotoxicity^18–20^. For example, propofol induced alteration of miRNA profile in the cultured neonatal rat astrocytes^21^. In stem cell-derived human neurons, our group identified that propofol-induced downregulation of miR-21 led to adverse mitochondrial health via the Sprouty 2 protein pathway^22^. Though these data shed some light, the complicated roles of various dysregulated miRNAs underlying propofol-induced neurotoxicity in the developing brain are not fully understood. Thus, the aim of this study was to investigate the acute and long-term effects of developmental propofol exposure on miRNA and mRNA profiles in the mouse hippocampus, and to utilize bioinformatic analysis to predict disease pathways, miRNA-mRNA interactions, and the related cellular/molecular events underlying neuronal endpoints (i.e., apoptosis, neurodegeneration, and cognitive function) affected by propofol in order to provide more clues for various mechanisms of the neurotoxic side effects of developmental propofol exposure.

## Results

### Propofol exposure does not affect physiological conditions in mice

No mouse pups died during propofol anesthesia. The body temperature was maintained at 37 ± 0.5 °C during propofol exposure. There were no significant differences in arterial blood gas parameters (Table 1) between control and propofol groups.

**Table 1:** Arterial blood physiological parameters in mice.

	Control	Propofol
pH	7.35 ± 0.02	7.31 ± 0.04
pCO_2_ (mm Hg)	43.93 ± 3.24	45.48 ± 2.75
pO_2_ (mm Hg)	84.83 ± 4.95	82.65 ± 5.40
HCO_3_^−^ (mEq/L)	22.53 ± 1.21	21.00 ± 1.65
tCO_2_ (mEq/L)	23.95 ± 1.28	21.38 ± 2.27

Note: pCO_2_: partial pressure of carbon dioxide;

pO_2_: partial pressure of oxygen; HCO_3_^−^: bicarbonate ion;

tCO_2_: total carbon dioxide.

### Propofol induces developmental neurotoxicity

**Figure 1:**
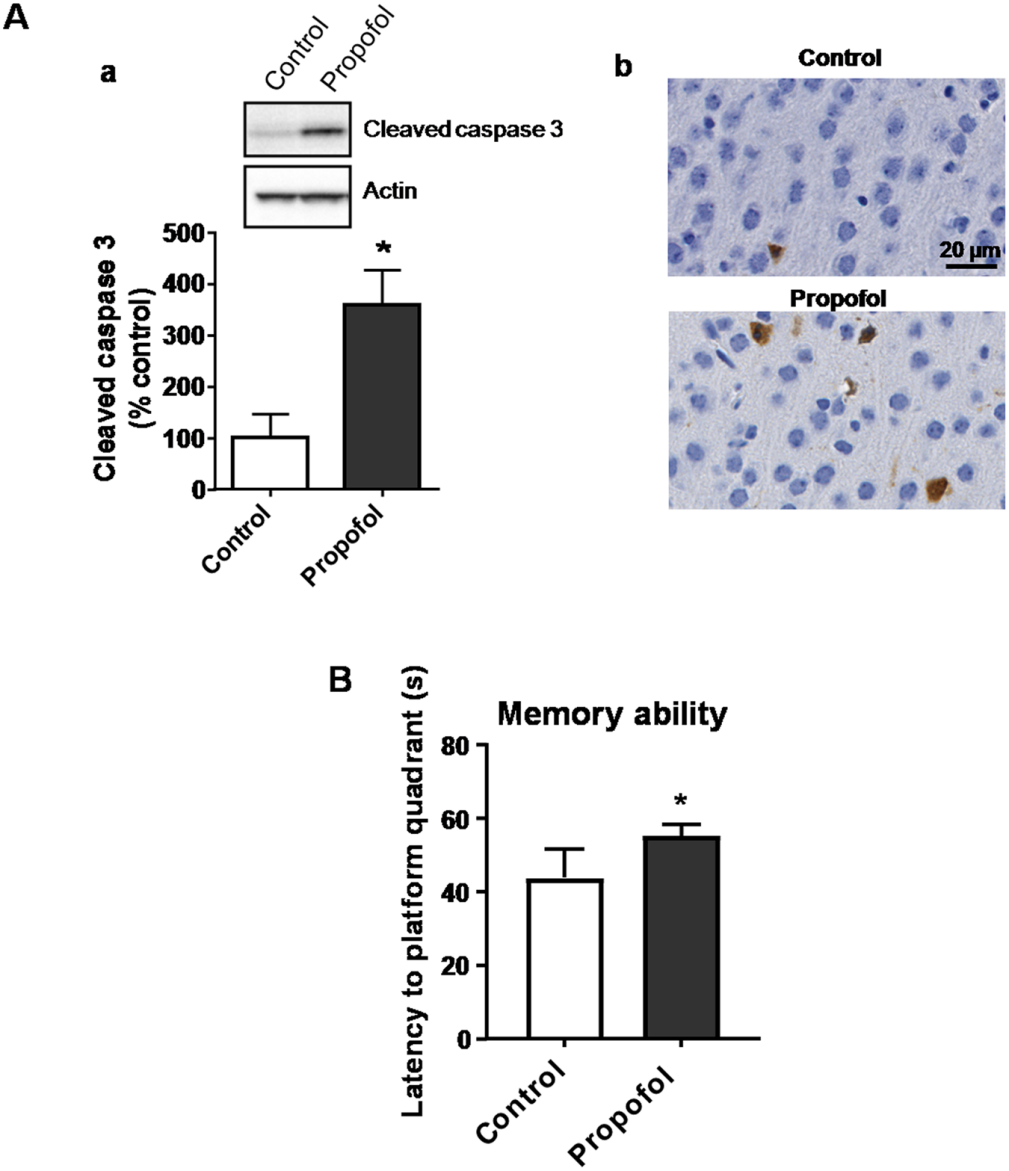
Propofol exposure for 3 hours induces acute apoptosis in the hippocampi of postnatal day 7 (P7) mice and impairs long-term memory function. (**A**) Apoptosis assay by evaluation of cleaved caspase 3expressionin the mouse hippocampus using Western blot and immunohistochemical staining. (a) Representative Western blot images were shown in the upper panel and quantification against actin was shown in the lower panel. The image of full-length membrane showing the protein signals of cleaved caspase 3 and actin from different samples was shown in Supplementary Fig. 1. (b) Representative immunohistochemistry images were shown for both groups. Brown signals indicate the apoptotic cells and blue are cell nuclei. Scale bar = 20 µm. (**B**) Memory function analysis using the Morris water maze test shows that P60 mice receiving neonatal propofol treatment took longer to reach the platform quadrant compared to the intralipid control mice. n = 4; *p < 0.05.

Propofol exposure for 3 hours induced an increase of cleaved caspase 3 (an apoptosis indicator) protein expression in the hippocampus of postnatal day 7 (P7) mice compared with mice exposed to the 10% intralipid vehicle control. Immunohistochemical images also displayed more cleaved caspase 3-positive apoptotic cells in the hippocampus (Fig. 1A). Morris Water Maze test showed that the time to reach the quadrant where the platform was located (a measurement of spatial information memory retention) was prolonged in the P60 mice receiving neonatal propofol exposure when compared to the intralipid-treated control group (Fig. 1B), suggesting that propofol treatment impaired long-term memory function.

### Propofol induces an alteration of mRNA profiles in the mouse hippocampus at P7

**Figure 2:**
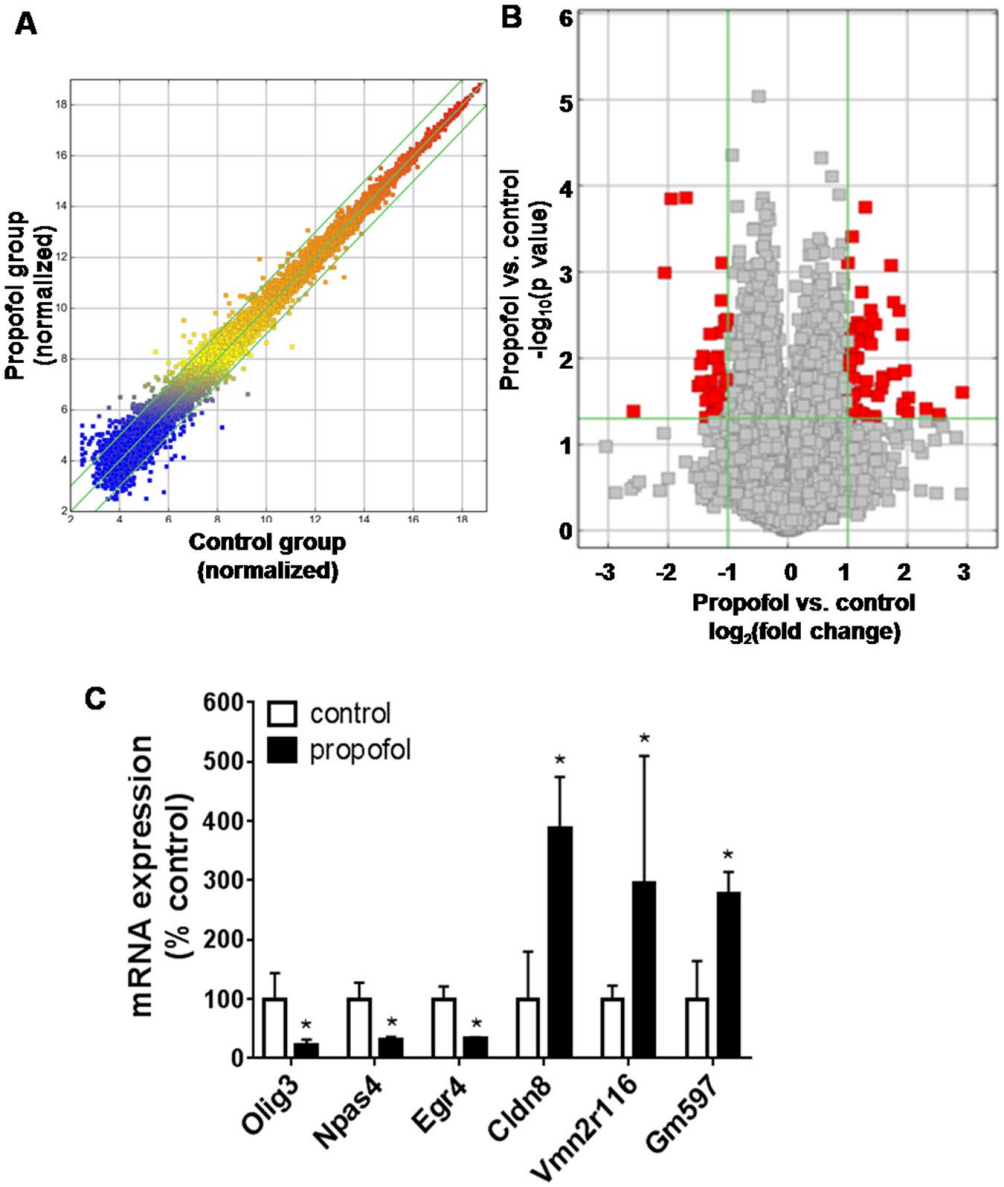
Propofol exposure results in the dysregulated mRNA profiles in P7 mouse hippocampi. (**A**) Scatter plot displaying that the overall mRNA transcriptome is similar between intralipid- and propofol-treated mouse hippocampal samples. (**B**) The volcano plot illustrating the differentially expressed mRNAs (red dots) between control and propofol groups. All mRNAs profiled were represented as points in the scatter plot with fold change and p value on the x and y axis, respectively. These altered mRNAs were either downregulated (left red dots) or upregulated (right red dots) following propofol exposure. (**C**) Reverse transcription-quantitative polymerase chain reaction (RT-qPCR) validation of propofol-induced changed mRNAs with top 3 highest up-regulated and top 3 down-regulated (including Olig3, Npas4, Egr4, Cldn8, Vmn2r116 and Gm597) according to the microarray assay. n = 4; *p < 0.05.

Scatter plot analysis demonstrated that intergroup correlations between normalized propofol and control mRNA expression profile on P7 mice were highly similar, suggesting that the overall transcriptome is similar between propofol-treated and control mouse hippocampi (Fig. 2A). Among 24,881 mRNA transcripts analyzed, there were 100 dysregulated mRNAs (67 upregulated and 33 downregulated) in the propofol-treated mouse hippocampi (fold change above ± 2, p < 0.05) at P7. The volcano plot illustrated the differentially abundant mRNAs in the hippocampi between control and propofol-treated groups (Fig. 2B). The mRNA expression level of the most robustly altered genes (3 up-regulated genes and 3 down-regulated genes: Olig3, Npas4, Egr4, Cldn8, Vmn2r116 and Gm597) were further validated using reverse transcription-quantitative polymerase chain reaction (RT-qPCR, p < 0.05; Fig. 2C), and the data were consistent with what was obtained from the microarray assay.

### Propofol induces an alteration of miRNA profiles in the mouse hippocampus at P7

**Figure 3:**
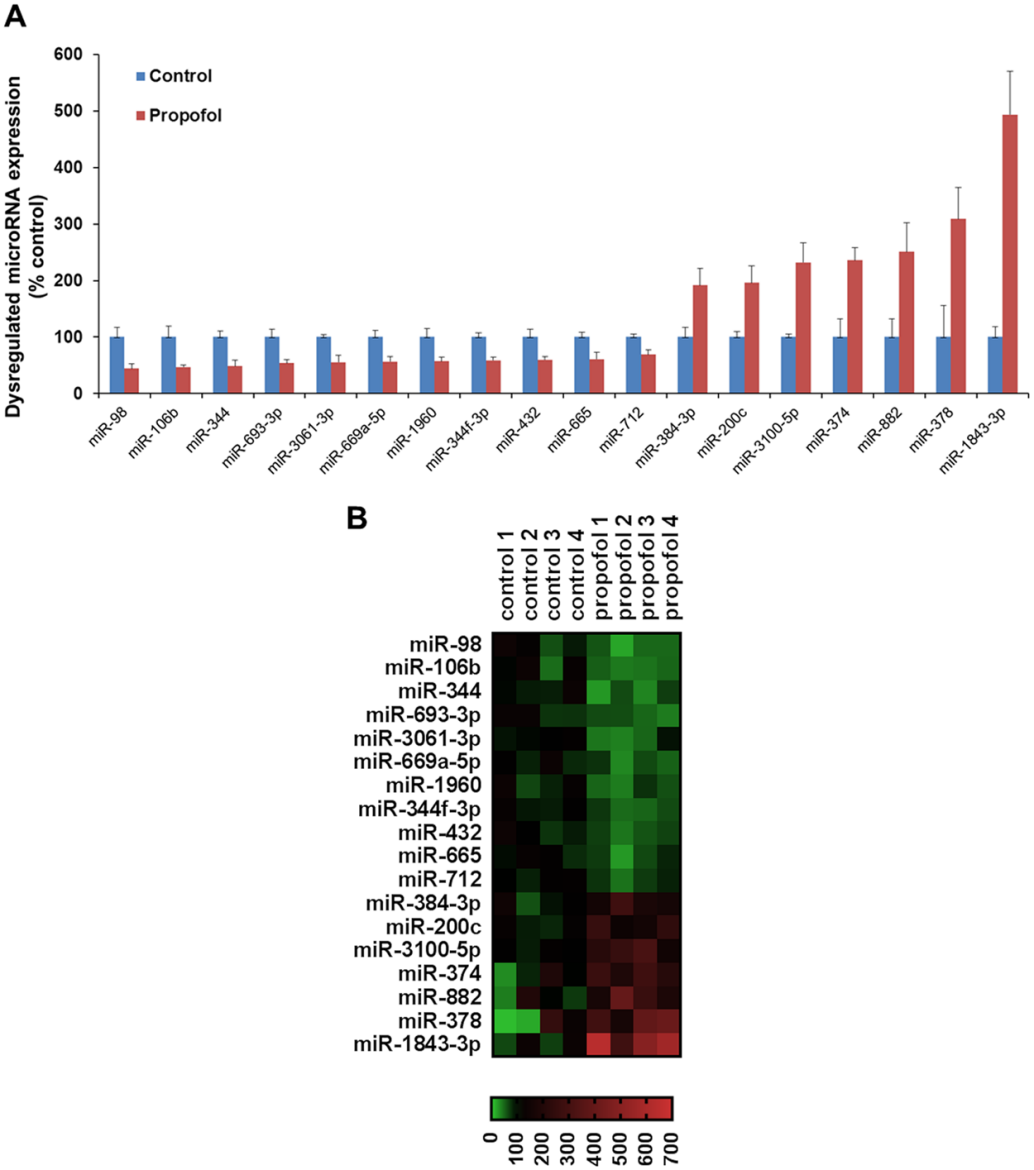
Propofol induces the dysregulation of miRNA profiles in 7-day-old mouse hippocampi. (**A**) Expression profile of altered miRNAs detected using RT-qPCR. miRNA expression in the propofol-treated group is expressed as percent of intralipid controls. (**B**) Heatmap showing the profiles of 18 differentially expressed miRNAs between intralipid and propofol-treated mouse hippocampi. The red, green, and black signals refer to the relatively higher, lower, and baseline levels of miRNA expression compared with the control group, respectively. Expression level of each sample for individual miRNAs was displayed as percentage of the control average. n = 4; p < 0.05.

Among the 726 mature mouse miRNAs analyzed using RT-qPCR assay, 699 miRNAs were detectable. The data showed that propofol increased the expression of 7 miRNAs (miR-384-3p, miR-200c, miR-3100-5p, miR-374, miR-882, miR-378, and miR-1843-3p) and decreased 11 miRNAs (miR-98, miR-106b, miR-344, miR-693-3p, miR-3061-3p, miR-669a-5p, miR-1960, and miR-344f-3p, miR-432, miR-665, and miR-712) in the P7 mouse hippocampi (p < 0.05 vs. intralipid vehicle control; Fig. 3A). The heatmap presents the relative expression patterns of the differentially expressed 18 miRNAs in control and propofol-treated individual mouse hippocampal samples (Fig. 3B).

### Potential signaling pathways for propofol-induced dysregulated miRNA involvement at P7

**Figure 4:**
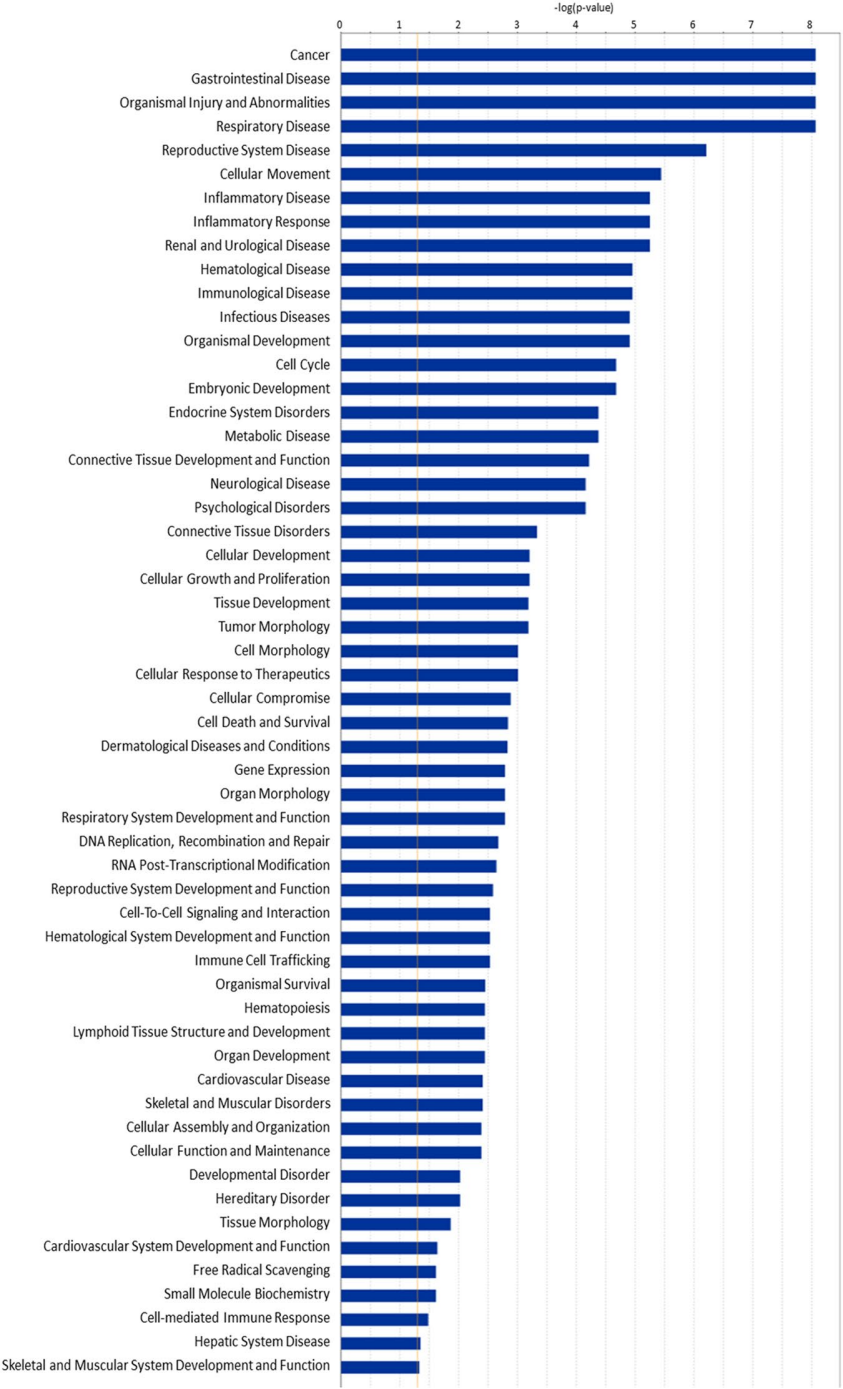
Bioinformatics analysis via Ingenuity Pathway Analysis indicates that propofol-induced dysregulation of 18 miRNAs in P7 mouse hippocampi are related to 56 signaling pathways. n = 4; p < 0.05.

Ingenuity Pathway Analysis (IPA) analysis shows that the propofol-induced dysregulated miRNAs on P7 are related to 56 signaling pathways (Fig. 4) which are involved in various diseases, development, and basic physiological processes. Specifically, some of these signaling pathways have been shown to be involved in neurological and psychological disorders (Alzheimer’s disease, primary multiple sclerosis and neuronal migration disorder), developmental disorders (Down syndrome), cell cycle (G1 phase), cellular growth and proliferation (cell proliferation of neuroblastoma cells), cellular compromise (degeneration of neuroblastoma cells), cell death and survival (apoptosis and necrosis), cellular assembly and organization (formation of actin stress fibers), organismal survival, gene expression (repression of mRNA), DNA replication, recombination and repair (chromosomal aberration, DNA damage), RNA post-transcriptional modification (deadenylation of mRNA), and free radical scavenging (production of hydrogen peroxide). These signaling pathways might participate in multiple propofol-induced side effects in neonatal mice.

### Dysregulated mRNA and miRNA interaction network at P7

**Figure 5:**
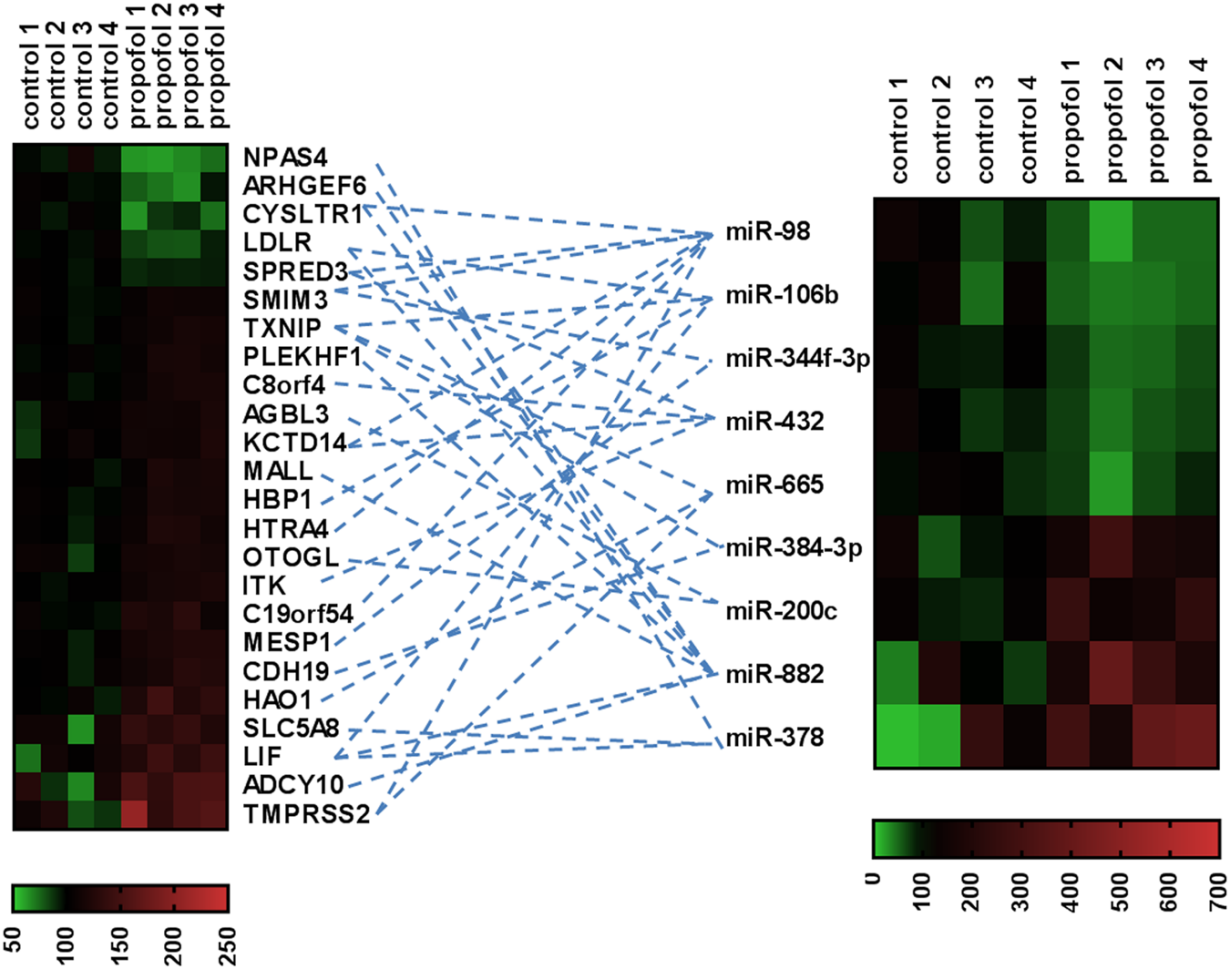
Interaction analysis of dysregulated miRNA-mRNA target pairs in P7 mouse hippocampi using Ingenuity Pathway Analysis. Among total propofol-induced 19 and 100 dysregulated miRNAs and mRNAs, respectively, 24 dysregulated mRNAs shown in the left heatmap might be the direct target genes of 9 dysregulated miRNAs displayed in the right heatmap. The blue lines indicate that those miRNAs putatively target the corresponding mRNAs. The red signal refers to the relatively higher expression, green signal referred to the relatively lower expression, and black refers to baseline of miRNA expression. Expression level of each sample for individual miRNAs was displayed as percentage of control average. There was an inverse relationship between the expression level of one miRNA and its target genes detected in the mouse hippocampus in response to the propofol treatment.

As described in the introduction, miRNAs negatively regulate their target mRNA by complementary binding of their seed sequence to the target mRNAs^13^. As a result, the target mRNA is degraded or the process of translation is repressed^14^. To find out the potential mRNA targets of the dysregulated miRNAs and the potential signaling involved in propofol-induced developmental neurotoxicity, we analyzed all propofol-induced dysregulated miRNAs and mRNAs using IPA to calculate the putative direct target mRNAs of the dysregulated miRNAs. The results displayed that there were 9 propofol-dysregulated miRNAs correlated with 24 putative target mRNAs altered following propofol exposure on P7. The 9 miRNAs and 24 mRNAs formed 34 miRNA-mRNA target interaction pairs (Fig. 5). These miRNA-mRNA interaction target pairs are mostly negatively correlated with each other. The dysregulated miRNA-mediated mRNA signaling pathway might participate in propofol-induced developmental neurotoxicity through multiple mechanisms (Table 2), such as apoptosis, abnormal morphology of astrocytes/cerebellum/nerve ending, cell injury, cellular migration, expansion of neural stem cells, growth of axons, myelination of oligodendrocytes, neurogenesis, reactive oxygen species, and release of Ca^2+^.

**Table 2:** The propofol-induced acute dysregulated miRNA-mediated mRNA signaling that potentially participate in propofol-induced developmental neurotoxicity in P7 mice.

Target mRNAs	miRNAs	Possible neurodegeneration-related pathways
NPAS4	miR-378a-3p	Apoptosis, Necrosis
ARHGEF6	miR-882	Apoptosis, Cell Migration, Necrosis
CYSLTR1	miR-98/882	Apoptosis, Cell Injury, Cell Migration, Necrosis, Release of Ca^2+^, Thickening of Brain Septum
LDLR	miR-106b/882	Apoptosis, Abnormal Morphology of Astrocytes/Cerebellum/Nerve Ending, Cellular Migration, Necrosis, Quantity of Reactive Oxygen Species
SPRED3	miR-98/432	Unknown
SMIM3	miR-98/344f-3p	Unknown
TXNIP	miR-106b/384-3p/665	Apoptosis, Cell Injury, Necrosis, Quantity of Reactive Oxygen Species
PLEKHF1	miR-882	Apoptosis, Necrosis
C8orf4	miR-432	Apoptosis, Necrosis
AGBL3	miR-200c	Unknown
KCTD14	miR-98/432	Unknown
MALL	miR-882	Unknown
HBP1	miR-106b	Unknown
HTRA4	miR-98	Unknown
OTOGL	miR-200c	Unknown
ITK	miR-432	Apoptosis, Cellular Migration, Necrosis, Release of Ca^2+^
C19orf54	miR-98	Unknown
MESP1	miR-344f-3p	Cell Migration, Development of Neuroectoderm, Migration of Stem Cell
CDH19	miR-384-3p	Unknown
HAO1	miR-665	Cell Migration
SLC5A8	miR-378a-3p	Apoptosis, Necrosis
LIF	miR-106b/378a-3p/882	Apoptosis, Abnormal Morphology of Cerebellum/Astrocytes, Cellular Migration, Expansion of Neural Stem Cells, Growth of Axon, Induction of Astrocyte/Neurons, Myelination of Oligodendrocytes, Necrosis, Neurogenesis of Neural Stem Cells, Proliferation of Neural Stem Cells, Quantity of Neural Stem Cells/Reactive Oxygen Species, Release of Ca^2+^
ADCY10	miR-882	Apoptosis, Cell Migration, Growth of Axon, Necrosis
TMPRSS2	miR-98/665	Unknown

### Dysregulated mRNA/miRNA profiles and potential interactive signaling at P60

**Figure 6:**
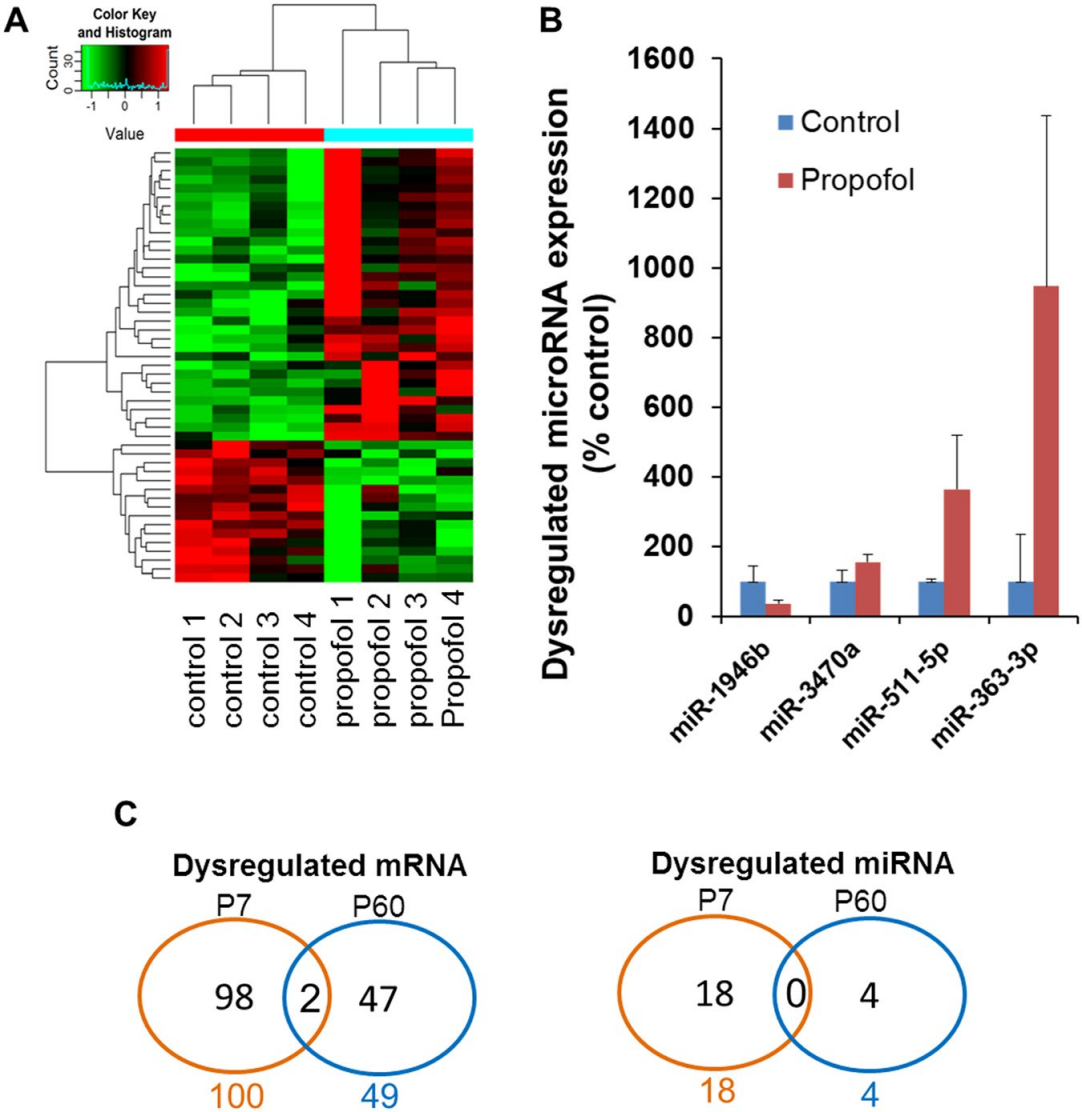
Propofol exposure results in dysregulated mRNA/miRNA profiles and interacting signaling in the P60 mouse hippocampus. (**A**) Hierarchical clustering of differentially expressed mRNA genes between control and propofol treated mouse hippocampi on P60 was displayed by heatmap (p < 0.05). The red signal denotes high relative expression and the green signal denotes low relative expression. (**B**) Expression profile of altered miRNAs detected using RT-qPCR (p < 0.05). miRNA expression in the propofol-treated group is expressed as percent of intralipid controls. (**C**) Venn diagrams illustrating numbers of differentially expressed mRNAs and miRNAs shared between P7 and P60. An overlap of 2 differentially expressed mRNAs but no differentially expressed common miRNAs between P7 and P60 mouse hippocampi were identified.

Neonatal propofol exposure resulted in long-term abnormal mRNA and miRNA profiles in mouse (P60) hippocampi. There were 49 dysregulated mRNAs (33 upregulated and 16 downregulated, fold change above ± 2, p < 0.05) (Fig. 6A) and 4 dysregulated miRNAs (3 upregulated and 1 downregulated, p < 0.05) (Fig. 6B) in P60 mouse hippocampi following neonatal propofol exposure. IPA analysis showed that one propofol-induced dysregulated miRNA was correlated with 2 putative target mRNAs that were altered in P60 mice following propofol exposure at P7. These altered miRNAs and mRNAs formed 2 miRNA-mRNA target interaction pairs (Fam46a/miR-363-3p and Rgs3/miR-363-3p). Bioinformatics analysis further indicates that the dysregulated miRNA-mediated mRNA signaling pathway might participate in propofol-induced long-term cognitive function through multiple mechanisms (Table 3). In addition, 2 differentially expressed mRNAs (Fam46a, Olfr961; fold change above ± 2, *p* < 0.05) were detected in both P7 and P60 mouse hippocampi, and this overlap over time was significantly related to treatment (p < 0.05; Fig. 6C). To explore non-significant but potentially important mRNAs or miRNAs (0.05 < p < 0.10 with no fold change limitation), we further summarized the Supplementary Tables 1 and 2. We found that there were 20 potentially important mRNAs (0.05 < p < 0.01) in both P7 and P60, but no candidate miRNAs commonly changed (p < 0.10) in both P7 and P60 stages.

**Table 3:** The propofol-induced long-term dysregulated miRNA/mRNA signaling that potentially participate in propofol-induced cognitive dysfunction in P60 mice.

mRNA/miRNA	Possible mechanism related to cognitive dysfunction
Rgs3 (target by miR-363-3p)	Chemoattraction of cerebellar granule cells, migration of cerebellar granule cells, axonal guidance signaling,
Fam46a (target by miR-363-3p)	Unknown
miR-363-3p	This microRNA has been reported to be associated with nonfamilial Alzheimer disease, Schizophrenia, Bipolar disorder

## Discussion

The present study is the first to explore the propofol-induced dysregulation of acute and long-term miRNA/mRNA expression profiles in hippocampal tissue from P7 mice from an interactive, signaling pathway perspective. We found that a sub-anesthetic dose of propofol induced acute neuroapoptosis in the neonatal mouse hippocampus and impaired long-term memory capacity in mice (Fig. 1). These propofol-induced acute neurotoxic effects were accompanied by the altered expression of 100 mRNAs and 18 miRNAs at P7 (Figs 2 and 3). These propofol-dysregulated miRNAs on P7 are significantly involved in various pathways such as neurological, psychological, developmental disorders, and organismal injury (Fig. 4). In particular, bioinformatics analysis shows that 24 dysregulated mRNAs might be the putative target genes of 9 abnormally expressed miRNAs, forming 34 miRNA-mRNA interaction target pairs (Fig. 5). Additionally, neonatal propofol also resulted in the long-term abnormal expression of 49 mRNAs and 4 miRNAs in P60 mice (Fig. 6). The 2 dysregulated mRNAs might be the putative target genes of one abnormally expressed miRNA, forming 2 miRNA-mRNA interaction target pairs. These both acute and long-term dysregulated miRNA-mRNA target pairs potentially contribute to propofol-induced pathological neurological and developmental disorder processes in mice (Tables 2 and 3).

Neuronal apoptosis has been one of the most widely studied neurodegenerative outcomes following developmental propofol exposure^23^. The current study assessed neuron apoptosis following a relatively short 3-hour exposure to propofol, suggesting that neuronal injury conferred by propofol rapidly initiates cell death early because of immediate cellular/molecular events. Considering the clinical relevance of the neurodegeneration we observed following propofol exposure, the memory function impairment (Fig. 1B) is of the greatest interest in conjunction with evidence of anesthesia in young children. Much remains unknown how these apoptotic early effects of propofol directly contribute to persistent cognitive dysfunction. In addition, multiple molecular networks are likely required to maintain neuronal integrity. By exploring the miRNA/mRNA interface and the related signaling networks utilizing bioinformatics analysis, our study has shed much light on this matter, and greatly informed future mechanistic studies in the field.

Of the 100 and 18 significantly dysregulated mRNAs and miRNAs altered by propofol on P7, respectively (Figs 2 to 5), it was predicted that 24 mRNA/miRNA interactions may be leading to the observed downstream apoptosis and cognition (Fig. 1), and other altered cellular and molecular events reported such as synapse loss and decreased dendritic spine density^24,25^. Among these dysregulated miRNAs, possible roles of a few (e.g., miR-200, miR-106b, and miR-278a) have been investigated previously in cell stress studies. For instance, the miR-200 family was increased in cypermethrin-induced neuron-like differentiated PC12 cells (a neurotoxicity cell model in developing rats), while the overexpression of miR-200b/c can lead to the down regulation of its target BCL2 protein and resultant increase in apoptosis^26^. Downregulation of the miR-106b-25 cluster was found to be crucially involved in endoplasmic reticulum stress-induced apoptosis and the upregulation of Bim in mouse embryonic fibroblasts and MCF-7 human breast cancer cells^27^. In our data, the increased miR-200c and downregulated miR-106b are consistent with the reports regarding their general role during cell apoptosis, suggesting these miRNAs might participate in propofol-induced developmental neurotoxicity through cellular apoptotic pathways. Moreover, there is one study working on miRNA expression profiling in propofol-treated hippocampal astrocytes from neonatal rats, and miR-378a-3p was significantly increased in both one hour-10 µg/ml propofol and 48 hour-0.9 µg/ml propofol treatment group^21^. We also found that miR-378a-3p to be overexpressed following propofol for 3-hour plus 3-hour wash out in the mouse neonatal hippocampus. In short, a few propofol-dysregulated miRNAs found in our study were previously mentioned in other developmental neurotoxicity studies with consistent findings, suggesting that these dysregulated miRNAs might have similar pathway involvement in propofol-induced cellular apoptosis signaling.

The bioinformatics analysis of the mRNA and miRNA expression profiling in P7 mice shows that 24 dysregulated mRNAs might be the putative target genes of 9 abnormally expressed miRNAs (Fig. 5). Our pathway analysis of propofol-dysregulated miRNAs and dysregulated miRNA-target mRNA interaction network identified pathways similar to what has been found to occur in other neurodegenerative diseases, such as Alzheimer’s disease, primary multiple sclerosis, and Down syndrome (Table 2). Other signaling pathways of propofol dysregulated miRNAs overlap with existing neurotoxicity pathways, such as cell cycle^28^, growth and proliferation^29^, cell apoptosis and necrosis^30^, neurogenesis^31^, mitochondrial dysfunction^32^, and calcium release^33–35^. These predictions are extremely useful as researchers are able to elucidate complex phenotypes, and pinpoint new targets affected by anesthetics. The pathway prediction also offered novel insights into other physiological processes which might contribute to the neurotoxicity, such as abnormal morphology of astrocytes/nerve endings, cell injury, cellular migration, chromosomal aberration, DNA damage, dysregulated neuronal migration, expansion of neural stem cells, formation of actin stress fibers, growth of axons, myelination of oligodendrocytes, neuron cell degeneration, organismal survival, production of hydrogen peroxide, reactive oxygen species, and repression and deadenylation of mRNA (Table 2). Specifically, miR-98 has an inverse relationship with oxidative stress in Alzheimer’s disease^36^, so its downregulation by propofol in our study may be contributing to oxidative stress-induced cellular consequences. Our analysis predicted that propofol-induced miR-98 downregulation may result in the upregulation of the expression of 7 mRNAs (CYSLTR1, SPRED3, SMIM3, KCTD14, HTRA4, C19orf54 and TMPRSS2) (Fig. 5), likely contributing to the apoptosis observed in this study, and other possible abnormal developmental events such as impaired cell migration.

Another interesting dysregulated miRNA is miR-665. It has been reported that propofol and several other anesthetic drugs (e.g., sevoflurane) mostly act through γ-Aminobutyric acid type A receptors^37^. Our data showed that propofol downregulated miR-665 expression (Fig. 3). Surprisingly, one recent publication reported that sevoflurane also downregulated miR-665 in neonatal rat brains. miR-665 mimics significantly increased its expression and reversed all the molecular changes and improved motor performance through PI3K/Akt signaling pathway by targeting insulin-like growth factor 2^38^. These data suggest that both propofol and sevoflurane might share similar miR-665 neurotoxic signaling pathways. As shown in the Fig. 5, there are three putative target genes of miR-665 that were upregulated following propofol exposure: TXNIP, HAO1, and TMPRSS2. Bioinformatics analysis indicates that TXNIP is related to apoptosis, cell injury, necrosis, and reactive oxygen species while HAO1 is involved in cell migration (Table 2). The functional roles of miR-665 and whether TXNIP, HAO1, and TMPRSS2 are the direct targets of miR-665 in propofol-induced developmental neurotoxicity in mice remain under future investigation.

In this study, we focused on both acute and long-term effect of propofol on the miRNA/mRNA profiles. The acute miRNA/mRNA profile on mice and pathway analysis were performed at an early time point (3 hours) following propofol exposure on P7, as the focus was pinpointing cellular/molecular pathways aberrantly regulated in the acute stages of propofol toxicity. Propofol-induced neurotoxicity is a complex physiological process, such that an early, single exposure to anesthetics can cause long-term deficits. In addition, just like the transient cellular and molecular events of propofol-induced toxicity have long-lasting impacts on behavior and function, it is likely that miRNAs/mRNAs and their corresponding pathways show upregulation and downregulation throughout various developmental days which may contribute to the progress of the neurotoxicity. Hence, we investigated the long-term effect of neonatal propofol exposure on miRNA/mRNA profile on P60 mice. According to our data, Fam46a and Olfr961 mRNAs were found dysregulated in the hippocampus of both P7 and P60 mice based on propofol exposure at P7. FAM46A (Family with sequence similarity 46, member A1) was originally implicated to be related to retinal neurodegeneration. A sequence variant of FAM46a, which was located very close to the initiation codon of this gene, was found in Spanish families with retinitis pigmentosa^39^. Interestingly, an exploratory genome-wide association study (GWAS) further indicates that a risk loci named rs72907046 which is near FAM46A could contribute to the vulnerability of posterior cortical atrophy in Alzheimer’s disease^40^. Olfr961 (olfactory receptor 961) was previously identified as one of eugenol-responsive odorant receptor in mice^41^, while its role related to neurodegeneration was not studied before. Four miRNAs including miR-1946b, miR-3470a, miR-511-5p and miR-363-3p and the potential Fam46a-miR-363-3p and Rgs3-miR-363-3p pairs were dysregulated on P60. Among them, there has been no implication in cognitive dysfunction except for miR-363-3p. miR-363-3p was previously found elevated in the hippocampus of patients with late-onset Alzheimer’s disease^42^, suggesting its strong implication in cognitive dysfunction. It was also highly expressed in the hippocampus from the propofol exposed P60 mice. Hence, our original findings indicated that the common dysregulated mRNAs (Fam46a and Olfr961) and the interacting target signaling (especially Fam46a-miR-363-3p pair) might play a crucial role in the development of neonatal propofol exposure-induced pathogenesis in the hippocampus and long-term cognitive dysfunction.

There is a limitation in this study regarding the equivalent exposure length between mice and humans. Most previously published rodent neurotoxicity studies used 2 to 6 hours of exposure length. We do not suggest direct over-interpretation of our data into human clinical practice due to various relative anesthetic exposure duration differences and different life spans between mice and human beings. It is also important to investigate the functional roles of these dysregulated genes in anesthetic-induced developmental neurotoxicity using developmental human neurons in future studies.

Collectively, our current data provide (1) evidence of acute neuroapoptosis and long-term behavioral dysfunction following a relatively short exposure to propofol in the developing mouse hippocampi, (2) neonatal propofol exposure-induced acute and long-term changes of miRNA and mRNA profiles, and (3) a detailed interaction network showing predictive pathways based on mRNA/miRNA interactions relevant to neurodegenerative phenotypes. Studying anesthetic-induced neurotoxicity has posed many challenges, including identifying intermediate players between anesthetic exposure and cellular endpoints. Obtaining the mRNA/miRNA profile and target network following anesthetic exposure is a novel concept. Insights on both known and lesser-known phenotypes resulting from developmental neurotoxicity conferred by propofol are extremely relevant. Our findings will inform future explorations in this field in teasing apart the functional contribution of the potential mRNA/miRNA pathways altered by the action of anesthetics and uncovering abnormal cellular endpoints.

## Methods

### Animal studies

All animal studies were approved by the Institutional Animal Care and Use Committee at the Medical College of Wisconsin. All methods were performed in accordance with the relevant guidelines and regulations. P7 C57BL/6 mice (Jackson laboratories, Bar Harbor, ME, USA) were used in the propofol exposure study. Experiments were performed in animals of both genders, and mice were randomly distributed into either the propofol or control groups.

### Anesthetic exposure

P7 mice received intraperitoneal injections of 50 milligram (mg) per kilogram (kg) propofol (2, 6-diisopropylphenol, C_12_H_18_O) (ZOETIS, Parsippany-Troy Hills, NJ) while the control mice were treated with the 10% intralipid (vehicle, Fresenius Kabi AB, Uppsala, Sweden). It was reported that single intraperitoneal injection of propofol maintained anesthesia in mice for approximately 90 minutes^9^. Hence, every 90 minutes, propofol/intralipid was administered again to maintain anesthesia during the entire 3 hours. The mice were placed on a heated pad (37 °C) in an open chamber provided with room air during the whole anesthetic exposure. The rectal temperature was periodically checked to ensure maintenance of body temperature at 37 ± 0.5 °C using a thermometer. Depth of anesthesia defined by the response to righting reflex and toe pinch was monitored during the procedure. Some pups were used for transcardial arterial blood gas analysis immediately after 3 hour-propofol exposure. Some mice were placed on the heated pad (37 °C) for another 3 hours after the anesthesia procedure, provided with room air during the wash out phase. After this 3-hour propofol exposure plus 3-hour wash out of propofol treatment, some mice were immediately euthanized for isolation of hippocampal tissues used for RNA and protein assay. Some mice treated with propofol were returned to home cages for cognitive function assay on P60, and then euthanized for isolation of hippocampal tissues for mRNA and miRNA profile assay.

### Western blot

Mouse hippocampal tissues on P7 were harvested and lysed in cell lysis buffer (Cell Signaling, MA) containing phosphatase inhibitor cocktails (Roche Diagnostics, Indianapolis, IN) for protein preparation. Total protein of 10 micrograms (μg) was loaded for Western blot assay. The primary antibodies used were rabbit anti-cleaved caspase 3 (Cell Signaling, Danvers, MA), and rabbit anti-actin (Cell Signaling, Danvers, MA). The secondary antibody used was anti-rabbit immunoglobulin G horseradish peroxidase-linked (Cell Signaling, Danvers, MA). The protein membranes were developed using an ECL Detection kit (GE Healthcare Life Sciences, Marlborough, MA) and signals were captured using a Chemidoc imaging system (Bio-Rad, Hercules, CA). Actin was used as a loading control during protein normalization of cleaved caspase 3.

### Immunohistochemical staining

P7 mice were subjected to perfusion-fixation using 10% zinc formalin fixation solution (Richard-Allan Scientific, San Diego, CA, USA) through the left ventricle once the animal reached a surgical plane of anesthesia with isoflurane. Afterward, the brains were quickly removed and further post-fixed with formalin overnight at room temperature. The brain tissues were cut into the right and left hemispheres along the anatomic midline. Paraffin-embedded brain hemisphere tissue blocks were then cut into 4 µm-thick sagittal sections. The cut position was around 600 µm distance to the midline. The sections were deparaffinized with xylenes, hydrated through graded ethanol, and then subjected to antigen retrieval by incubation in target retrieval solution (Dako, Santa Clara, CA, USA) for 20 minutes in a boiling steamer. Then, sections were washed three times with phosphate buffered saline (PBS) containing 0.5% Triton X-100 (Sigma, Milwaukee, WI, USA), and blocked with 10% donkey serum (Thermo Fisher Scientific, Waltham, MA, USA) for 30 minutes at room temperature. The sections were stained with primary antibody rabbit anti-cleaved caspase 3 (apoptosis marker; Cell Signaling) in a humidified chamber for 30 minutes at 37 °C. After three washes, slides were incubated sequentially with HRP polymer conjugate and DAB chromogen provided in the SuperPicture™ Polymer Detection Kit as described in the manual provided by the company (Thermo Fisher Scientific). The slides were finally counterstained with Hematoxylin. The coverslips were mounted on glass slides with mounting medium (Thermo Fisher Scientific). The brain sections were photographed using a microscope (Olympus Corporation of the Americas, Center Valley, PA, USA).

### Morris water maze

The Morris water maze assay was performed to assess the effect of propofol on spatial memory retention of P60 mice receiving propofol or intralipid injection at P7 as previously described^43^. Briefly, a white circular polypropylene pool (100 cm in diameter and 20 cm in height) was filled with water plus a food coloring agent, rendering it opaque. On the pool rim, four points were designated (north, east, south, and west), dividing the pool into four quadrants (NE, NW, SW, SE). The pool water was changed and the temperature was checked daily to be 20–22 °C. Platforms were 8 × 8 cm positioned at the center of the SE quadrant, with the standing area submerged ∼5 cm below the surface of the water. The mouse was positioned in the water from random start points and ended when the mouse reached and escaped onto the hidden platform. The platform was in the SE quadrant every day and the mouse was given 4 trials per day with an interval of 5 minutes for 6 days. If unable to find the platform in 60 seconds, the mouse was guided by the investigator. On the 7^th^ day, the memory assay was performed. The platform was removed from the pool. The mouse was positioned in the water from a random start points and ended when the mouse reached and escaped to SE quadrant. EthoVision XT (Noldus Information Technology, Washington, US), the most widely applied video tracking software, was used to track and analyze the behavior, movement, and activity of any animal. Escape latency (indicating memory function) on 7^th^ day was calculated based on time spent to escape to the SE quadrant. At the end of experiments, all animals were euthanized by carbon dioxide inhalation.

### RNA extraction

Total RNA was isolated using the phenol-chloroform method as previously described^22^. Briefly, after mouse hippocampal tissues were lysed in the QIAzol lysis reagent (Qiagen Inc., Valencia, CA), chloroform was thoroughly mixed with the samples, set for 5 minutes before centrifuged. Supernatants were transferred into a new tube and mixed with isopropanol and centrifuged again for pellets. After washing with 75% ethanol for one time, the pellets were dried and dissolved in RNase free water. The quantity and purity of RNA were validated using a Nanodrop spectrophotometer (Thermo scientific, Waltham, MA, US).

### mRNA profiling assay

Mouse mRNA Expression Microarray V3.0 assay and data analysis were performed by Arraystar Inc. (Rockville, MD) to evaluate global expression levels of mRNAs in hippocampi using the purified RNA. Prior to the microarray assay, RNA quality control (QC) analysis of RNA quantity, purity, integrity, genomic DNA contamination, and label efficiency was validated using Agilent Technologies following the GE1_1100Jul11 protocol. Criteria for detailed statistical analysis for the mRNA microarray quality control are available through Arraystar (www.arraystar.com). The RNA was converted to complementary DNA (cDNA), and hybridized to the 24,881mRNA. P value was calculated using unpaired t-test, while FDR is calculated from Benjamini Hochberg FDR. Fold Change was calculated using the absolute ratio of the normalized intensities between two conditions. Similar distribution of overall mRNA transcriptome between control- and propofol-treated mouse hippocampal samples was illustrated in scatter plot. The significantly differentially expressed mRNAs were defined by the expression level above ±2.0-fold change and p < 0.05 between propofol and control samples, and are shown in volcano plots or heatmaps. The volcano plot is constructed by plotting the negative log of the p value on the y axis (base 10). The x axis is the log of the fold change between the propofol and control conditions. The heatmap shows the gene profile for all samples, with red expressing most highly up-regulated and green expressing most highly down-regulated genes.

### miRNA profiling assay

miRNA profiling assay was performed using mouse miRNA qPCR profiling array (Applied Biological Materials Inc./Qiagen). cDNA of hippocampi was used as templates for analysis of total 726 mature mouse miRNAs by RT-qPCR. Altered miRNAs were defined based on p < 0.05 between propofol and control groups.

### Reverse transcription-Quantitative PCR (RT-qPCR)

mRNA microarray assay was further validated by RT-qPCR to confirm the mRNA expression profile results obtained from the microarray. The 6 mRNAs which were most robustly modified by propofol based on the mRNA microarray assay were analyzed by qPCR. Among these 6 mRNAs, 3mRNAs (Cldn8, Vmn2r116 and Gm597) were up regulated and 3 mRNAs (Olig3, Npas4 and Egr4) were down regulated following propofol expression. cDNA (1 µg) was synthesized using an RT^2^ First Strand Kit (Qiagen) following manufacturer’s instruction. For the PCR assay, cDNA was mixed with Power up SYBR Green Master Mix (Thermo Fisher Scientific), primers, and pure water (Qiagen). Triplicate samples were loaded into 384-well plates (10 μl/well) and PCR was performed using QuantStudio™ 6 Real-Time PCR detection system (Thermo Fisher Scientific) for 10 minutes at 95 °C followed by 40 cycles of a denaturation step (15 seconds at 95 °C) and a combined annealing/extension step (30 seconds at 60 °C). The mean cycle threshold (Ct) values of triplicate wells for each sample were collected and the expression data was normalized to the endogenous control (beta-actin). Melting curves were monitored to validate the purity of the PCR product in each well. The primer sequences are listed as follows: Olig3: forward 5′-GGCGATATGGTCCAGAAGATGC-3′, reverse 5′-GTCCGTTGATCTTCAGCCGCAA-3′; Npas4: forward 5′-TCTTGCCTGCATCTACACTCGC-3′, reverse 5′-TCCAGGTAGTGCTGCCACAATG-3′, Egr4: forward 5′-TTCTCTCCAAGCCCACCGAA-3′, reverse 5′-AGCTCAAGAAGTCGCCTCCA-3′, Cldn8: forward 5′-GTGGAGAGTGTCTGCCTTCATC-3′, reverse 5′-TAAGAGCCAGCAGGGAGTCGTA-3′, Vmn2r116: forward 5′-CAGCAGACAGTTGTGAATGGG-3′, reverse 5′-AAGGGCCAGTATACATTTGGTT-3′, Gm597: forward 5′-AGTCACCACCATCTGGGAGCAA-3′, reverse 5′-GGCTTTCCACAGGCCATATTGC-3′.

### IPA of differentially expressed mRNAs, miRNAs, signaling pathway and their interaction networks

The IPA software (Qiagen Bioinformatics, CA) was chosen to analyze the potential mechanism and canonical physiological signaling pathways of differentially expressed miRNAs, and the dysregulated miRNA-mRNA target interaction in this study. The potential signaling pathways in propofol-induced developmental neurotoxicity was nominated by input of all 19 differentially expressed miRNAs into the IPA database (p < 0.05 compared with control group). All significant related pathways (with Fisher’s exact test p < 0.05) were displayed and studied in this work. miRNA-mRNA interaction was analyzed by IPA miRNA target filter module which calculate the putative miRNA-mRNA target interaction relationship using Targetscan database with input of all propofol-induced dysregulated miRNAs and mRNAs. The above-mentioned miRNA-interacting mRNAs were further analyzed for possible signaling pathways related to neurodegeneration.

### Statistical analysis

The data were presented as mean ± standard deviation (SD) from 4 intralipid control and 4 propofol-treated mice. For statistical analysis of differentially expressed mRNA and miRNAs and Western blotting, Student’s t-test or Mann-Whitney test was chosen for analysis of the differences between two groups. Shapiro-Wilks test was then used for validation of the normal distribution of the data, and Levene test was used for the validation of variance homogeneity. To test whether overlap between differentially expressed mRNA and miRNA at P7 and P60 was significantly related to treatment or based on chance, the Fisher’s Exact Test was performed. Analysis of dysregulated miRNAs-mRNA signaling pathways was described in the above sections. SPSS software (IBM SPSS Statistics Version 24, US) was used for data analysis and p < 0.05 was considered statistically significant. Besides, all commonly changed mRNA or miRNAs (p < 0.10 without fold change limitation in both P7 and P60 array) were further explored and summarized in Supplementary Tables 1 and 2. This will inform the readers that the genes with statistically non-significant but potentially important trends (e.g., 0.05 < p < 0.1) in one or the other stage have the common changes (p < 0.1) in both stages.

## Electronic supplementary material

Supplementary Materials

## Data Availability

The datasets generated during and/or analyzed during the current study are available on the NCBI data base, with the GEO Submission Number GSZ106799.
