# Supplementary material for: Signaling network between the dysregulated expression of microRNAs and mRNAs in propofol-induced developmental neurotoxicity in mice
Source: Sci Rep. 2018 Sep 21;8:14172. doi: 10.1038/s41598-018-32474-3 (PMC6155049; doi:10.1038/s41598-018-32474-3)
Supplement: Supplementary file 1 — Supplementary Materials [file 41598_2018_32474_MOESM1_ESM.pdf]

Signaling network between the dysregulated expression of microRNAs and mRNAs in propofol-induced developmental neurotoxicity in mice

Congshan Jiang[1,2,#], Sarah Logan[3,4,#], Yasheng Yan[3], Yasuyoshi Inagaki[1], Thiago Arzua[3,4], Peizhong Ma[1], Shemin Lu[2], Zeljko J. Bosnjak[4,5] & Xiaowen Bai[3,4,]

[1]Department of Anesthesiology, Medical College of Wisconsin, Milwaukee, WI, USA

[2]Department of Biochemistry and Molecular Biology, School of Basic Medical Science, Xi'an Jiaotong University Health Science Center, Xi'an, Shaanxi, China

[3]Department of Cell Biology, Neurobiology & Anatomy, Medical College of Wisconsin, Milwaukee, WI, USA

[4]Department of Physiology, Medical College of Wisconsin, Milwaukee, WI, USA

[5]Department of Medicine, Medical College of Wisconsin, Milwaukee, WI, USA

[#]Contributed equally to this work

Supplementary Figure 1

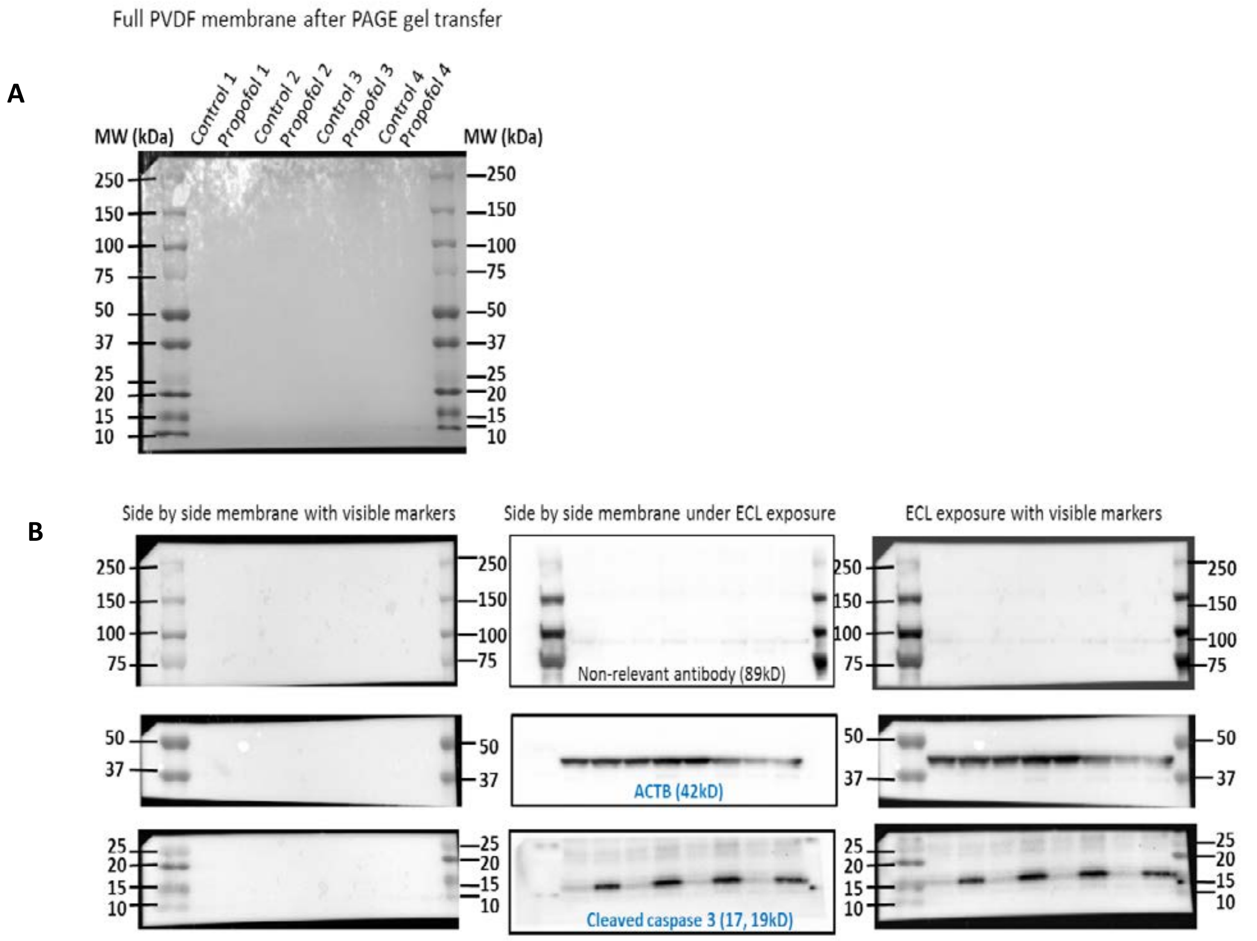

Supplementary Figure 1: (A) The image of full-length membrane of Figure 1A-a. Protein lysates from mouse hippocampi were collected after 3 h propofol exposure and 3 h wash out. The effect of propofol on the cleaved caspase 3 (apoptosis marker) levels in mouse hippocampus was analyzed by Western blot. Following gel running and membrane transferring, the membrane was probed with antibodies against cleaved caspase 3 (apoptosis marker) and beta-actin (ACTB, endogenous control). n=4 for both control and propofol mouse groups. (B) The images of the membrane showing the signals of beta-actin and cleaved caspase 3.

Supplemental table 1. Side by side comparison of the effect of neonatal propofol exposure-induced abnormally expressed mRNAs (with P<0.10) in postnatal day 7 (P7) and P60 mouse hippocampus

	P7			P60		
**GeneSymbol**	**P-value**	**Fold Change**	**Regulation**	**P-value**	**Fold Change**	**Regulation**
Egr4	0.00	3.86	down	0.08	1.20	up
Pdk4	0.01	2.09	up	0.02	1.38	up
BC024978	0.01	2.53	up	0.04	1.65	up
Plekhf1	0.01	2.26	up	0.04	1.65	up
Smim3	0.01	2.06	up	0.07	1.20	up
Aldh1a3	0.01	2.06	up	0.02	1.70	up
Prss53	0.02	2.05	down	0.03	1.42	up
Cysltr1	0.02	2.42	down	0.05	1.39	up
Olfr961	0.03	3.82	up	0.01	2.22	up
Fam46a	0.04	2.39	down	0.00	2.54	up
Tnfaip3	0.05	2.31	down	0.04	1.84	up
Olfr960	0.05	4.93	up	0.00	2.54	up
Cndp1	0.05	2.37	up	0.06	3.35	up
Crebl2	0.07	2.02	up	0.06	1.10	up
Aga	0.09	2.29	up	0.04	1.71	up
Filip1	0.08	1.30	up	0.00	3.33	down
Lgr6	0.08	1.26	down	0.02	2.34	down
Olfr401	0.06	1.98	up	0.03	2.15	down
Brd8	0.05	1.19	down	0.03	2.04	down
Styk1	0.06	1.45	up	0.03	2.53	down
Taf9b	0.04	1.43	down	0.05	2.37	up
Drd3	0.09	2.24	down	0.07	2.21	down

Note: Different color areas represent different genes with different P values.

P7	P60
p < 0.05	p < 0.05
0.05 < p < 0.10	0.05 < p <0.10
Not shown if p > 0.10 or not detected	

Supplemental table 2. Side by side comparison of the effect of neonatal propofol exposure-induced abnormally expressed miRNAs (with P<0.10) in postnatal day 7 (P7) and P60 mouse hippocampus

	P7			P60		
**miRNA Symbol**	P-value	Fold Change	Regulation	P-value	Fold Change	Regulation
mmu-miR-1843-3p	0.00	4.93	up	0.93	1.08	down
mmu-miR-344f-3p	0.01	1.69	down	0.87	1.15	down
mmu-miR-3100-5p	0.01	2.32	up	0.40	2.47	down
mmu-miR-374	0.01	2.36	up	0.59	1.31	up
mmu-miR-344	0.01	2.05	down	0.52	1.36	up
mmu-miR-3061-3p	0.01	1.80	down	0.91	1.07	up
mmu-miR-200c	0.02	1.96	up	0.21	6.53	up
mmu-miR-712	0.02	1.45	down	0.85	1.10	up
mmu-miR-669a-5p	0.02	1.77	down	0.92	1.09	down
mmu-miR-98	0.03	2.25	down	0.75	1.34	down
mmu-miR-106b	0.03	2.12	down	0.99	1.01	down
mmu-miR-384-3p	0.04	1.92	up	0.47	1.42	up
mmu-miR-378	0.04	3.09	up	0.63	1.28	up
mmu-miR-665	0.04	1.64	down	0.79	1.13	down
mmu-miR-1931	0.05	1.83	up	0.35	2.83	down
mmu-miR-181a	0.06	4.66	down	0.95	1.06	down
mmu-miR-3103	0.06	2.59	down	0.66	1.52	down
mmu-miR-375	0.06	1.93	down	0.86	1.13	up
mmu-miR-3078	0.06	1.44	down	0.95	1.06	down
mmu-miR-3102-3p.2	0.06	1.49	down	0.62	1.34	up
mmu-miR-466j	0.06	1.57	down	0.96	1.03	up
mmu-miR-3077	0.07	2.27	up	0.98	1.02	down
mmu-miR-670	0.08	2.02	down	0.95	1.06	down
mmu-miR-3082-3p	0.08	1.36	down	0.89	1.12	down
mmu-miR-384-5p	0.08	1.54	down	0.47	1.42	up
mmu-miR-673-5p	0.08	1.32	down	0.16	2.67	up
mmu-miR-379	0.08	1.76	down	0.51	1.97	down
mmu-miR-1-2-as-3p	0.08	4.67	up	0.90	1.09	up
mmu-miR-664	0.08	3.46	down	0.86	1.10	up
mmu-miR-3084	0.08	1.45	up	0.94	1.06	down
mmu-miR-449a	0.08	1.73	up	0.55	1.81	down
mmu-miR-1193-3p	0.10	1.58	up	0.99	1.01	up
mmu-miR-511-5p	0.48	2.08	down	0.02	3.64	up
mmu-miR-1946b	0.78	1.28	down	0.03	2.68	down
mmu-miR-363-3p	0.44	2.68	up	0.04	9.47	up
mmu-miR-3470a	0.34	2.95	down	0.05	1.54	up
mmu-miR-669i	0.56	1.34	up	0.07	2.74	up
mmu-miR-147	0.97	1.01	up	0.09	1.81	up
mmu-miR-196b	0.99	1.01	up	0.09	68.38	down
mmu-miR-450b-5p	0.12	5.35	up	0.09	3.35	up

Note: Different color areas represent different genes with different P values.

P7	P60
p < 0.05	p < 0.05
0.05 < p < 0.10	0.05 < p <0.10
p > 0.10	
